# OSM Enhances Angiogenesis and Improves Cardiac Function after Myocardial Infarction

**DOI:** 10.1155/2015/317905

**Published:** 2015-06-04

**Authors:** Xiaotian Zhang, Di Zhu, Liping Wei, Zhijing Zhao, Xin Qi, Zongjin Li, Dongdong Sun

**Affiliations:** ^1^Department of Cardiology, Xijing Hospital, Fourth Military Medical University, 127 West Changle Road, Xi'an, Shaanxi 710032, China; ^2^Department of Cardiology, Tianjin Union Medicine Center, Tianjin 300121, China; ^3^Department of Pathophysiology, Nankai University School of Medicine, 94 Weijin Road, Tianjin 300071, China

## Abstract

Oncostatin M (OSM) has been reported to stimulate angiogenesis by upregulating VEGF and bFGF, implying that it could be a therapeutic strategy in treating ischemic diseases. The present study was aimed at investigating whether OSM could improve cardiac function via prompting angiogenesis following myocardial infarction (MI). Wild type (WT) and O*β* knock-out (O*β* ^−/−^) mice were, respectively, randomized into sham group, MI + vehicle group, and MI + OSM group. WT mice displayed significantly impaired cardiac function after MI. OSM treatment attenuated cardiac dysfunction in WT MI mice, while O*β* deletion abrogated the protective effects. Besides, OSM attenuated heart hypertrophy and pulmonary congestion evidenced by decreased heart weight/body weight and lung weight/body weight ratio. Further, reduction of apoptosis and fibrosis in infarct border zone was observed in OSM treated WT MI mice compared with vehicle. Moreover, in WT mice subjected to MI, OSM treatment significantly increased capillary density along with upregulation of p-Akt and angiogenic factors VEGF and bFGF in comparison with vehicle, and this phenomenon was not found in O*β* ^−/−^ mice. In conclusion, OSM treatment preserved cardiac function, inhibited apoptosis and fibrosis, and stimulated angiogenesis via upregulating VEGF and bFGF in infarct border zone of ischemic myocardium, indicating that OSM could be a novel therapeutic target for MI.

## 1. Introduction

Myocardial infarction (MI) remains one of the major causes of mortality and disability worldwide [1, 2]. Loss of cardiomyocytes in the infarction area and remodeling of the spared myocardium result in cardiac dysfunction [3]. Although MI triggers a spontaneous angiogenic response which could reestablish myocardial blood flow, this protective response is usually insufficient to restore the physiological level of perfusion [4]. Hence, therapeutic induction of angiogenesis is recognized to be a valid approach to restore the supply of oxygen and nutrients to the ischemic regions and improve compromised cardiac function [5, 6].

Oncostatin M (OSM) is a member of the interleukin-6 (IL-6) cytokine family produced by inflammatory cells. Murine OSM binds to its receptor O*β*, which exists as a part of a heterodimer with the glycoprotein 130 (gp130) signal transducer, and exerts multiple physiological functions. Previous studies have reported that OSM is involved in regulating cell proliferation, differentiation, survival, regeneration, and remodeling of various tissues [7]. Gwechenberger et al. reported that OSM contributed to the process of cardiomyocytes repair after myocardial infarction [8] and Pöling et al. have confirmed that blocking OSM signaling attenuates inflammatory dilative cardiomyopathy [9]. Besides, OSM also has the ability to upregulate some potent angiogenic factors, such as vascular endothelial growth factor (VEGF) [10], basic fibroblast growth factor (bFGF) [11, 12], and angiopoietin2 (Ang2) [13]. Whether OSM could induce angiogenesis in the heart after myocardial infarction thus restoring myocardial perfusion remains largely unknown.

Therefore, the aim of the study was to establish the role of OSM in enhancing VEGF and bFGF production, reducing cardiomyocytes fibrosis and apoptosis, and improving cardiac function after myocardial infarction.

## 2. Methods

### 2.1. Experimental Animals

Animal experiments were performed following the guidelines in accordance with the National Institutes of Health Guidelines on the Use of Laboratory Animals and were approved by the Fourth Military Medical University Ethic Committee on Animal Care. 129-Osmrtm1.1Nat/J mice were purchased from Jackson Laboratory (Bar Harbor, Maine, USA) which possess loxP sites on both sides of the second exon (first coding exon) of the oncostatin M receptor (O*β*) gene. 129-Osmrtm1.1Nat/J mice were crossed with C-Tg (CMV-cre) 1 Cgn/J mice (Jackson Laboratory, USA) to knockout oncostatin M receptor O*β*. PCR analysis was used to screen O*β* ^−/−^ mice, and O*β* ^+/+^ mice (weight 23–25 g) were used as control. Mice were randomly allocated into six groups: (1) sham (WTS); (2) myocardial infarction (MI) + vehicle (WTMI); (3) WTMI + OSM; (4) O*β* ^−/−^ sham (O*β* ^−/−^S); (5) O*β* ^−/−^MI + vehicle (O*β* ^−/−^MI); (6) O*β* ^−/−^MI + OSM. MI was induced by permanent left anterior descending (LAD) coronary artery ligation. Sham groups underwent identical surgical procedure without coronary ligation.

### 2.2. Surgical Procedures

Mice were anesthetized with 3% isoflurane, then orally intubated with a 22G IV catheter, and ventilated with a rodent respirator (Harvard Apparatus, Hilliston, USA). MI was produced by temporarily exteriorizing the heart via a left thoracic incision and placing a 6-0 silk suture around the left anterior descending coronary artery. The lungs were inflated by positive end-expiratory pressure and the chest was closed with 6.0 nylon suture. After surgery, the animals were weaned from the respirator and then placed on a heating pad for recovery. Sham mice underwent the same surgical procedures except for the fact that the suture placed under the left coronary artery was not tied.

### 2.3. OSM Treatment Protocol

OSM-treated mice received OSM injection after operation. OSM (R&D Systems, Inc., Minneapolis, MN, USA) was dissolved in sterile PBS containing 0.1% bovine serum albumin and injected intraperitoneally twice a day with 60 ng per gram of body weight for 14 days. Vehicle group received the same volume of sterile PBS containing 0.1% bovine serum albumin as for 14 days.

### 2.4. Echocardiography for Determination of Cardiac Function

Echocardiography was conducted 4 weeks after MI. Mice were sedated using 3% isoflurane and transthoracic echocardiography was performed by Vevo 2100 ultrasound system (Visual-Sonics, Toronto, Canada) with a 30 MHz linear transducer. Left ventricular ejection fraction (EF), left ventricular fractional shortening (FS), diastolic left ventricular internal diameter (LVIDd), and systolic left ventricular internal diameter (LVIDs) were calculated by the use of computer algorithms. All measurements were calculated based on the statistics of 4 consecutive cardiac cycles and all of these measurements were performed in a blinded manner [14].

### 2.5. Determination of Apoptosis

Heart samples were fixed in 4% paraformaldehyde and then paraffin embedded at day 14 as previously described [15]. Then, the hearts were cut into 5 *μ*m sections. Terminal deoxynucleotidyl transferase-mediated dUTP nick-end labeling (TUNEL) staining was carried out using a commercially available kit according to the manufacturer's instructions (Promega, Madison, WI, USA). Nuclei were stained by DAPI (Roche). Ten fields in the infarct border zone were randomly selected from each section for the calculation of the percentage of apoptotic nuclei (apoptotic nuclei/total nuclei) and the obtained ratios were averaged for statistical analysis.

### 2.6. Determination of Cardiac Fibrosis

To determine cardiac fibrosis of the heart, paraffin embedded sections (4 *μ*m) of the heart were stained by Masson's trichrome staining. In brief, the sections were deparaffinized in histo-clear and rehydrated using sequential passage through 100 to 70% ethanol for 6 min each followed by washing in distilled water three times. The slides were then stained with Weigert's iron hematoxylin for 10 min and washed under tap water for 10 min. The sections were washed again in distilled water and then stained with Biebrich scarlet-acid fuchsin solution for 15 min, in phosphomolybdic-phosphotungstic acid solution for 15 min and aniline blue solution, and stained for 10 min. The sections were rinsed briefly in distilled water and were treated with 1% acetic acid solution for 5 min. After a final wash in distilled water, the sections were dehydrated through sequential gradient of 70–100% alcohol followed by histo-clear wash and then mounted using Permount. The heart tissue sections were digitally imaged in high pixel resolution on an Epson Scanner.

### 2.7. Quantitative Real-Time PCR

RNA was isolated by using a NucleoSpin RNA II kit (Macherey-Nagel GmbH) and cDNA prepared with a Reverse Transcription System kit (Promega Corp). Quantitative real-time polymerase chain reaction was performed using predesigned Taqman Gene Expression Assays and AmpliTaq Gold DNA polymerase following the manufacturer's instructions (Applied Biosystems Inc.). The ratio of the mRNA levels for each sample was calculated by normalizing the comparative quantitation values to those of GAPDH mRNA. PCR was performed in a GeneAmp PCR system 2400 Thermal Cycler (Perkin-Elmer, Norwalk, CT, USA). Primers used were as follows: GAPDH, 5′-ACG GCA AAT TCA ACG GCA CAG TCA-3′ (forward) and 5′-TGG GGG CAT CGG CAG AAG G-3′ (reverse); Collagen I, 5′-TGC CGT GAC CTC AAG ATG TG-3′ (forward) and 5′-CAC AAG CGT GCT GTA GGT GA-3′ (reverse); Collagen III, 5′-AGA TCA TGT CTT CAC TCA AGT C-3′ (forward) and 5′-TTT ACA TTG CCA TTG GCC TAG-3′ (reverse).

### 2.8. Determination of Capillary Density

Capillary density was performed 14 days after the surgical procedure by immunohistochemistry. Mice were sacrificed, hearts were removed, and paraffin-embedded sections were prepared. Endothelial cells were labeled using goat polyclonal anti-CD31 (Santa Cruz Biotechnology, Santa Cruz, CA) followed by a biotinylated horse anti-goat secondary antibody (Vector Laboratories). The reaction product (brown) was visualized with 3,3′-diaminobenzidine substrate using the Vector ABC Vectastain Elite Kit (Vector Laboratories, Burlingame, CA). Images were captured and stored in digital Tiff file format for later image analysis. Counts of capillary and arteriolar density per square millimeter were obtained after superimposing a calibrated morphometric grid on each digital image using Adobe Photoshop Software.

### 2.9. Western Blot Assay

Protein was isolated from homogenized heart tissue following standard protocols. Total proteins were loaded onto an SDS-PAGE gel and transferred electrophoretically to nitrocellulose membranes (Millipore, Billerica, MA). After blocking with 5% skim milk, the membranes were incubated with the appropriate primary antibody of the recommended dilution at 4°C overnight. The membranes were washed and further incubated with horseradish peroxidase—linked secondary antibody at 37°C for 60 min. The blots were developed with an enhanced chemiluminescence reagent kit (Millipore, Billerica, MA) and visualized with UVP Bioimaging Systems. Vision Works LS Acquisition and Analysis Software were used to analyze blot densities.

Primary antibodies used in this study were as follows: OSM (R&D systems, Minneapolis, MN), O*β* (R&D systems, Minneapolis, MN), cleaved caspase 3 (Cell Signaling, USA), caspase 3 (Cell Signaling, USA), *β*-actin (Cell Signaling, USA), p-Akt (Cell Signaling, USA), Akt (Cell Signaling, USA), VEGF (R&D systems, Minneapolis, MN), and bFGF (UBI, Lake Placid, NY). Secondary antibodies were horseradish peroxidase-conjugated goat anti-rabbit IgG (R&D systems, Minneapolis, MN) and rabbit anti-goat IgG (R&D systems, Minneapolis, MN).

### 2.10. Statistical Analysis

Continuous variables that approximated the normal distribution were expressed as means ± standard deviation (SD). Comparisons between groups were subjected to ANOVA followed by Bonferroni correction for post hoc *t*-test. Data expressed as proportions were assessed with a Chi-square test. Two sided tests have been used throughout, and *P* < 0.05 was considered statistically significant. SPSS software package version 14.0 (SPSS, Chicago, IL) was used for data analysis.

## 3. Results

### 3.1. OSM Treatment Improved Cardiac Function after MI

**Figure 1:**
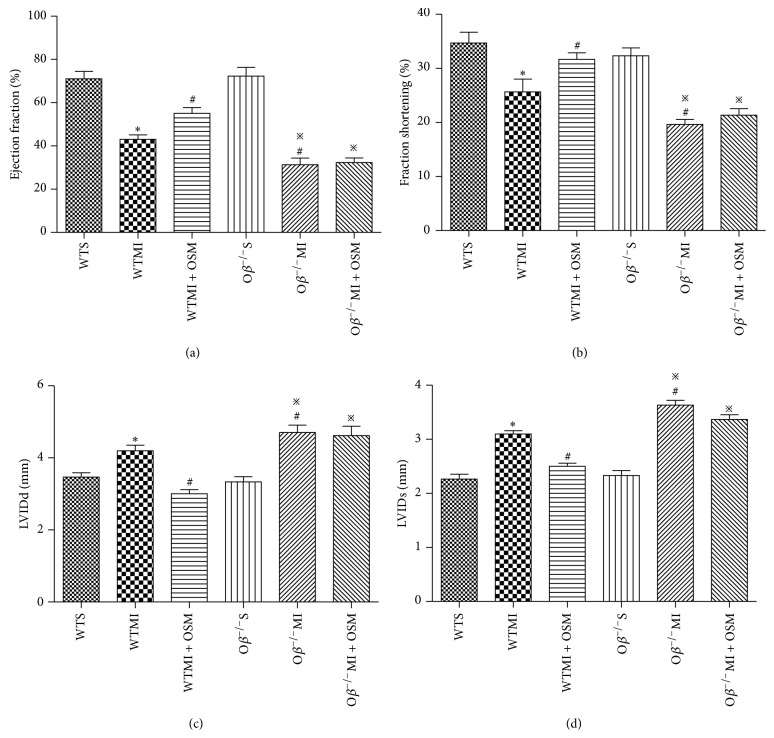
OSM treatment improved cardiac function after MI. Echocardiographic analysis of cardiac (a) ejection fraction, (b) fraction shortening, (c) left ventricular internal diastolic diameter (LVIDd), and (d) left ventricular internal systolic diameter (LVIDs) in the six groups of mice 28 days after operation. The values are means ± SD. ^*^ *P* < 0.05 versus WT group, ^#^ *P* < 0.05 versus WT + MI group, and ^*※*^ *P* < 0.05 versus O*β* ^−/−^S group. *N* = 18 in each group.

To determine whether OSM treatment could improve cardiac function in MI mice, echocardiographic investigations were carried out at day 28 after operation. In WT mice, MI induced significant cardiac function deterioration, evidenced by decreased EF value (WTMI versus WTS: 43.25 ± 2.12 versus 71.37 ± 3.49%, Figure 1(a)) and FS value (WTMI versus WTS: 25.37 ± 2.33 versus 34.67 ± 2.03%, Figure 1(b)) and increased LVIDd and LVIDs (Figures 1(c) and 1(d)). Deletion of O*β*, which abrogated OSM signaling, did not influence normal cardiac function before MI but resulted in a significant decline of left ventricular function after operation compared with WTMI mice (EF: O*β* ^−/−^MI versus WTMI: 31.24 ± 3.12 versus 43.25 ± 2.12%; FS: O*β* ^−/−^MI versus WTMI: 19.67 ± 0.88 versus 25.37 ± 2.33%, Figures 1(a) and 1(b)). Furthermore, a major improvement of cardiac function was observed when WTMI mice were treated with OSM (EF: WTMI + OSM versus WTMI: 55.38 ± 2.68 versus 43.59 ± 2.12%; FS: WTMI + OSM versus WTMI: 31.67 ± 1.20 versus 25.93 ± 2.33%, Figures 1(a) and 1(b)). However, the protective effect of OSM on cardiac function after MI was inhibited in O*β* knockout mice, indicating that OSM exerted cardiac protection in MI through O*β* receptor.

### 3.2. OSM Prevented Cardiac Hypertrophy in Response to MI

**Figure 2:**
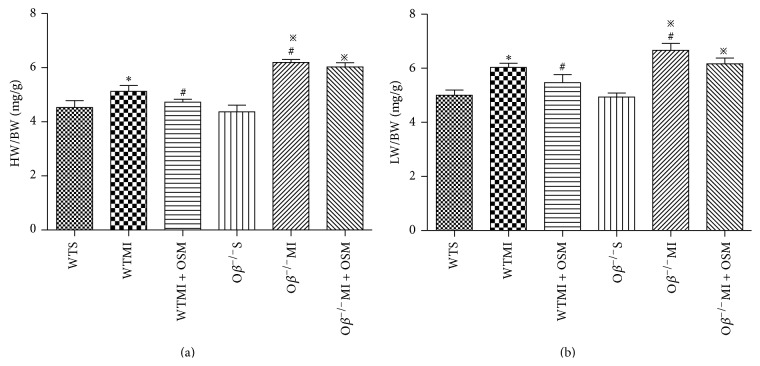
OSM prevented cardiac hypertrophy in response to MI. (a) HW/BW ratio in mice from all groups 14 days after MI. (b) LW/BW ratio in mice from all groups 14 days after MI. The values are means ± SD. ^*^ *P* < 0.05 versus WT group, ^#^ *P* < 0.05 versus WT + MI group, and ^*※*^ *P* < 0.05 versus O*β* ^−/−^S group. *N* = 18 in each group.

In order to investigate cardiac hypertrophy and pulmonary congestion, heart weight/body weight (HW/BW) ratio and lung wet weight/body weight (LW/BW) ratio were calculated 14 days after MI surgery. HW/BW ratio in WTMI mice was significantly increased compared with WT mice (WTMI versus WTS: 5.13 ± 0.12 versus 4.53 ± 0.15 mg/g, Figure 2(a)), and the change was reversed by OSM treatment (WTMI + OSM versus WTMI: 4.73 ± 0.06 versus 5.13 ± 0.12 mg/g, Figure 2(a)). However, O*β* deletion abrogated the beneficial effect of OSM (Figure 2(a)). Likewise, the increased LW/BW ratio in WTMI mice was reversed by OSM treatment (WTMI versus WTS: 6.03 ± 0.09 versus 5.00 ± 0.12 mg/g; WTMI + OSM versus WTMI: 5.47 ± 0.18 versus 6.03 ± 0.09 mg/g, Figure 2(b)), and the effect of OSM was inhibited in O*β* ^−/−^ mice (Figure 2(b)).

### 3.3. OSM Treatment Attenuated Cardiomyocyte Apoptosis after MI

**Figure 3:**
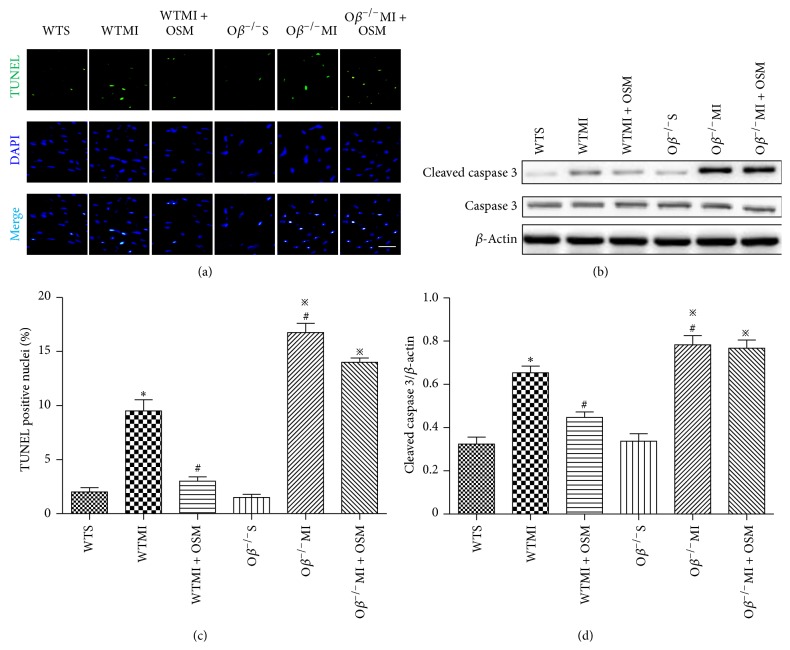
OSM treatment attenuated cardiomyocyte apoptosis after MI. (a) Representative digital micrographs showing cardiomyocyte apoptosis in the border zone of all groups of mice. Bar: 25 *μ*m. (b) Western blot assay of cleaved caspase 3 and caspase 3 in the border zone of the six groups of mice. (c) Quantitative analysis of cardiomyocyte apoptosis after MI in counts/high-power field (HPF). (d) Quantitative data of the expression of cleaved caspase 3 in the six groups of mice. The values are means ± SD. ^*^ *P* < 0.05 versus WT group, ^#^ *P* < 0.05 versus WT + MI group, and ^*※*^ *P* < 0.05 versus O*β* ^−/−^S group. *N* = 6 in each group.

The effect of OSM on the survival of myocardium in response to ischemia was evaluated at postoperative day 14 using TUNEL staining method. The ratio of apoptotic cardiomyocytes was significantly increased in WTMI mice compared with WT group, and this change was attenuated by OSM treatment (WTMI versus WTS: 9.52 ± 1.04 versus 2.00 ± 0.41%; WTMI + OSM versus WTMI: 3.27 ± 0.41 versus 9.52 ± 1.04%, Figures 3(a) and 3(c)). O*β* knockout MI mice demonstrated more severe cardiomyocyte apoptosis than WTMI mice (O*β* ^−/−^MI versus WTMI: 16.75 ± 0.85 versus 9.52 ± 1.04%), and OSM treatment no longer exerted the function of decreasing cardiomyocytes' susceptibility to ischemia damage (O*β* ^−/−^MI + OSM versus O*β* ^−/−^MI: 15.29 ± 1.08 versus 16.75 ± 0.85%, Figures 3(a) and 3(c)). Western blot analysis showed that the expression of cleaved caspase 3 was consistent with the extent of apoptosis as determined by the TUNEL staining (Figures 3(b) and 3(d)).

### 3.4. Postischemic Myocardial Interstitial Fibrosis Was Prevented by OSM Administration

**Figure 4:**
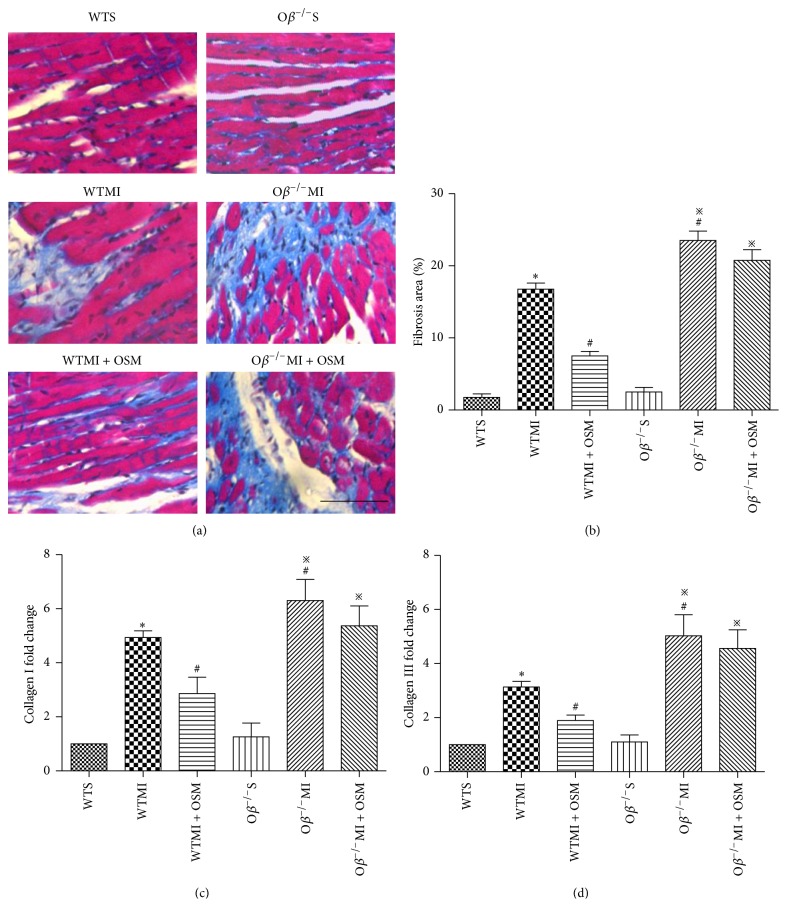
Postischemic myocardial interstitial fibrosis was prevented by OSM administration. (a) Representative images of Masson's trichrome-stained heart sections in all groups of mice 14 days after operation. Bar: 50 *μ*m. (b) Quantitative analysis of myocardial interstitial fibrosis in all groups. (c) mRNA fold change of the expression of Collagen I. (d) mRNA fold change of the expression of Collagen III. The values are means ± SD. ^*^ *P* < 0.05 versus WT group, ^#^ *P* < 0.05 versus WT + MI group, and ^*※*^ *P* < 0.05 versus O*β* ^−/−^S group. *N* = 6 in each group.

14 days after MI, myocardial interstitial fibrosis in the border zone was detected by Masson's trichrome staining and quantified with percentage of fibrosis area. WTMI mice induced an increase of fibrosis area in comparison with WT mice (WTMI versus WTS: 16.75 ± 0.85 versus 1.75 ± 0.48%, Figures 4(a) and 4(b)). O*β* knockout MI mice revealed even severe cardiomyocyte fibrosis compared with WTMI mice (O*β* ^−/−^MI versus WTMI: 23.50 ±1.32 versus 16.75 ± 0.85%, Figures 4(a) and 4(b)). OSM treatment resulted in remarkable reduction of fibrosis area in WT mice subjected to MI (WTMI + OSM versus WTMI: 7.52 ± 0.65 versus 16.75 ± 0.85%, Figures 4(a) and 4(b)), while it did not decrease fibrosis area in O*β* deletion MI mice. mRNA expressions of Collagens I and III in the border zone were consistent with the extent of fibrosis area as determined by Masson's trichrome staining (Figures 4(c) and 4(d)).

### 3.5. Treatment with OSM Promoted Angiogenesis by Increasing Capillary Density after MI

**Figure 5:**
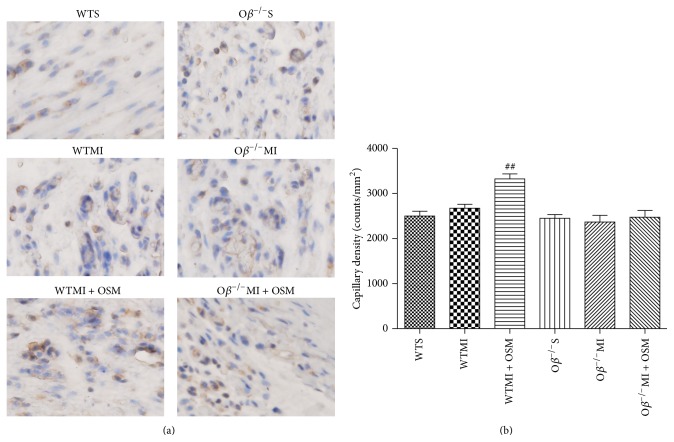
Treatment with OSM promoted angiogenesis by increasing capillary density after MI. (a) Representative digital micrographs showing capillary density/CD31 immunostaining 14 days after surgical intervention in different experimental groups. Magnification: 200x. (b) Quantitative analysis of capillary density in counts/mm^2^. The values are means ± SD. ^##^ *P* < 0.01 versus WT + MI group. *N* = 6 in each group.

The role of OSM treatment on the degree of angiogenesis following myocardial infarction was measured by capillary density through anti-CD31 immunohistochemistry staining 14 days after surgical intervention. WTMI mice revealed a minor increase of capillary density (WTMI versus WTS: 2675 ± 85 versus 2500 ± 108, *P* = 0.25), while OSM treatment significantly increased the capillary density (WTMI + OSM versus WTMI: 3325 ± 111 versus 2675 ± 85, Figures 5(a) and 5(b)). Conversely, no significant increase of capillary density was found in O*β* ^−/−^MI mice in comparison with the O*β* ^−/−^S mice and OSM treatment could no longer enhance angiogenesis as those detected in WT mice (O*β* ^−/−^MI versus O*β* ^−/−^S: 2370 ± 146 versus 2450 ± 84; O*β* ^−/−^MI + OSM versus O*β* ^−/−^MI: 2475 ± 149 versus 2370 ± 146, Figures 5(a) and 5(b)).

### 3.6. OSM Upregulated p-Akt, VEGF, and bFGF in Ischemic Myocardium

**Figure 6:**
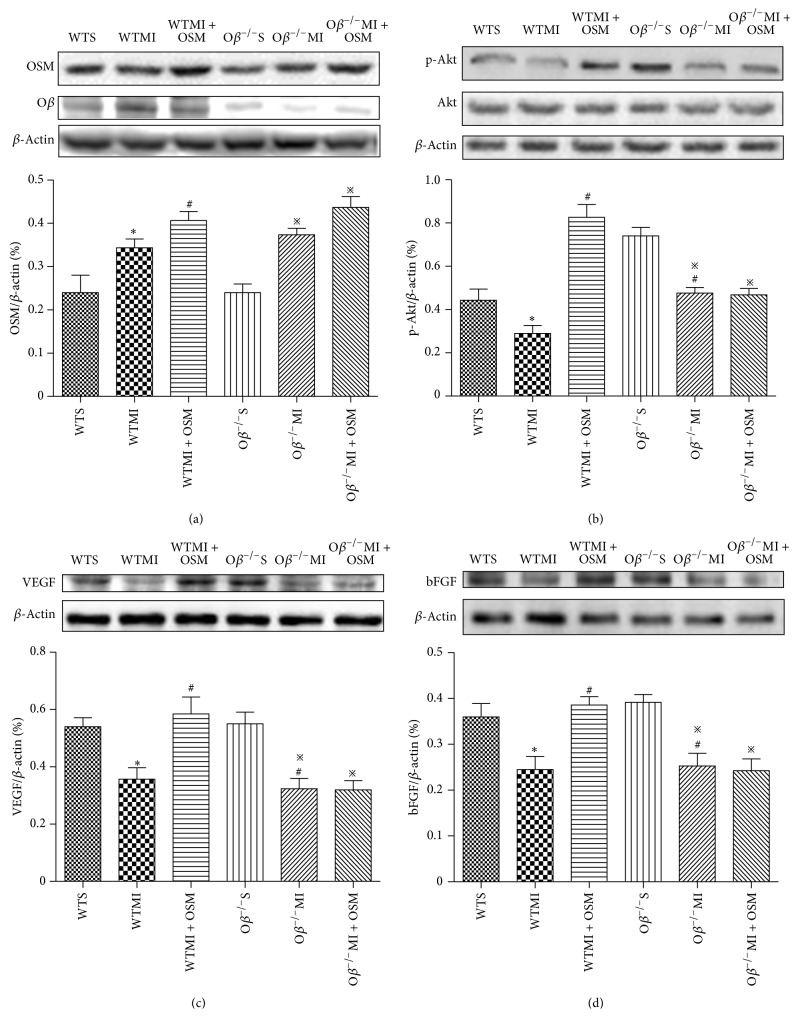
OSM increased levels of p-Akt, VEGF, and bFGF in ischemic myocardium. (a) Western blot analysis of the expressions of OSM in different groups of mice. (b) Western blot analysis of the levels of p-Akt and Akt in six groups. (c) Western blot analysis of the expression of VEGF in different groups of mice. (d) Western blot analysis of the expression of bFGF in the six groups of mice. The values are means ± SD. ^*^ *P* < 0.05 versus WT group, ^#^ *P* < 0.05 versus WT + MI group, and ^*※*^ *P* < 0.05 versus O*β* ^−/−^S group. *N* = 6 in each group.

Finally, we investigated the possible mechanisms responsible for the angiogenic effect of OSM during the process of myocardial infarction. 14 days after operation, MI group revealed increased OSM expressions in infarct border zone, which were even higher in OSM-treated groups (Figure 6(a)). WTMI mice revealed decreased levels of both p-Akt and VEGF compared with WT mice, and the changes were reversed by OSM treatment (Figures 6(b) and 6(c)). Decreased p-Akt and VEGF levels were observed in O*β* ^−/−^MI mice compared with O*β* ^−/−^S mice, while OSM treatment could not reverse the alteration as those detected in WT mice (Figures 6(b) and 6(c)). Another well-known angiogenic factor bFGF was investigated 14 days after operation. Similarly, expression of bFGF was decreased in WT mice after the MI surgery, and OSM treatment could reverse the change in WT mice, while nonattenuation was found in O*β* KO mice (Figure 6(d)). Taken together, Akt/VEGF and bFGF were both involved in the angiogenic effect of OSM during myocardial infarction.

## 4. Discussion

Our present study revealed that (1) OSM treatment preserved cardiac function and attenuated myocardial apoptosis and fibrosis in a mouse MI model; (2) OSM promoted angiogenesis in the infarct border zone after MI probably via upregulating angiogenic factors VEGF and bFGF; (3) the beneficial effect of OSM in MI mice was abrogated when OSM receptor O*β* was knocked out.

After LAD ligation, myocardial apoptosis is increased as a result of ischemia, and the lost myocardial mass is replaced by fibrosis due to the fact that myocardium has limited regenerative abilities. Thereafter, therapies which can increase angiogenesis and the blood supply to tissue are highly desirable [5, 14, 16]. Despite some progress that has been made in the past two decades, these proangiogenic treatments have, until today, not resulted in routine clinical applications [17]. VEGF is one of the most widely used angiogenic factors in previous studies [18]. Numerous experimental reports demonstrated the proangiogenic activity and the associated benefit on cardiac function of VEGF administration in porcine or rodent models of MI [13, 19]. Nevertheless, VEGF alone stimulates the formation of immature, leaky, and disorganized blood vessel, and this phenomenon emphasizes the need of timely regulated angiogenic signal for the formation of a stable and functional microvascular network capable of restoring blood flow to ischemic tissues [20]. bFGF is another potent angiogenic factor which has been reported to induce angiogenesis in the ischemic heart and promoted cardiac repair after MI [17]. Interestingly, Cao et al. reported that bFGF-induced angiogenesis leads to the formation of stable and mature blood vessels in the mouse cornea [21], contrary to VEGF-stimulated angiogenesis. The knowledge brought the idea that a combination of VEGF and bFGF might be a better therapy to induce angiogenesis following MI.

The inflammatory cytokine OSM has been reported to increase VEGF expression in various tissue types and upregulate endothelial bFGF expression* in vitro*, so we hypothesized that it might induce mature vessels after MI. In the present study, we demonstrated for the first time that OSM induced angiogenesis by upregulating expressions of both VEGF and bFGF in a MI mouse model, thus restoring blood flow of ischemic heart and reducing apoptosis and fibrosis due to less damage of cardiomyocytes. VEGF is an endogenous proangiogenic factor that can be induced immediately after MI due to a spontaneous angiogenic response of the heart, but the protein at the border zone is only increased during day 1 after MI. At day 14, VEGF level is lower than that before MI [22]. We observed in this study that supplement of OSM stimulated VEGF expression till 14 days after operation, indicating that OSM treatment might support the insufficient spontaneous angiogenic process induced by MI. The beneficial effect of OSM treatment was confirmed by improvement of cardiac function 28 days after operation, which was consistent with previous studies reported by Kubin et al. [23].

There are two types of OSM receptors, namely, type I receptor consisting of LIFR (leukemia inhibitory factor receptor-) gp130 and type II receptor formed by a complex of OSM receptor O*β* and gp130 [24]. Murine OSM binds exclusively to type II receptor while human OSM has the exceptional capability to recruit both receptors. In the mouse MI model exploited in the present study, O*β* knockout completely abrogated protective effects of OSM following operation, which further confirmed that OSM exerted its angiogenic function in MI mice through and merely through type II receptor.

## 5. Conclusions

The present study provided direct evidence that OSM increased cardiac function and inhibited apoptosis and fibrosis through inducing angiogenesis via upregulating VEGF and bFGF in a mouse MI model. These findings suggested that OSM might be a promising therapeutic tool in treating MI.
